# Black Tongue: A Rare Presentation of Rhupus Syndrome

**DOI:** 10.7759/cureus.40240

**Published:** 2023-06-11

**Authors:** Piyush Puri, Princy Sardana, Ninia Goyal, Rajpreet S Arora, Akshit Chitkara

**Affiliations:** 1 Internal Medicine, Rama Medical College Hospital and Research Center, Hapur, IND; 2 Internal Medicine, Saraswathi Institute of Medical Sciences, Hapur, IND; 3 Internal Medicine, Chirayu Medical College and Hospital, Bhopal, IND; 4 Internal Medicine, Uttar Pradesh University of Medical Sciences (UPUMS), Etawah, IND; 5 Internal Medicine, University of California Riverside School of Medicine, Los Angeles, USA

**Keywords:** black hairy tongue, auto-immune like, rhupus syndrome, sle and rheumatoid arthritis, rheumatoid arthriitis

## Abstract

Black tongue is a benign condition typically caused by the overgrowth of dead skin cells, resulting in elongated papillae and a hairy appearance. Other factors contributing to this condition include inadequate oral hygiene, a soft diet, and staining from bacteria, food, yeast, and other substances. It may cause symptoms such as bad breath, a metallic taste in the mouth, and an unsightly black hairy-looking tongue.

Here, we present a case of a 30-year-old female who came to our hospital complaining of bad breath and a black tongue for the past month. She had previously taken antibiotics at the primary care medical center, but there was no improvement. We then prescribed her fluconazole, an antifungal medication, for the next two weeks. After two weeks, she returned with a slightly improved tongue color. Further investigation revealed a history of abortion and mild intermittent joint pains, for which she had been self-medicating with over-the-counter acetaminophen. A complete work-up led to the discovery of positive anti-cyclic citrullinated peptide (anti-CCP) antibodies, anti-double-stranded (anti-DS) DNA antibodies, and ANA, leading to the diagnosis of Rhupus Syndrome, an overlap of systemic lupus erythematosus (SLE) and rheumatoid arthritis (RA).

## Introduction

Rhupus syndrome, coined by Peter Schur in 1971, refers to a rare condition where patients meet the criteria for both systemic lupus erythematosus (SLE) and rheumatoid arthritis (RA) [1]. In Rhupus syndrome, SLE-related features are generally mild and primarily involve the skin, mucosa, and blood [2]. While there are no specific diagnostic criteria for Rhupus syndrome, the established standards for diagnosing SLE (SLICC-2012) and RA (ACR/EULAR-2010) are used. Additionally, joint surface erosion, high levels of RA factor, anti-cyclic citrullinated peptide (anti-CCP) antibodies, ANA, and anti-double-stranded (anti-DS) DNA are considered in identifying Rhupus syndrome [3]. Due to the rarity of this condition, limited information is available regarding its pathophysiology, prevalence, natural history, immunological characteristics, and radiological findings. The exact cause and triggers of Rhupus syndrome remain largely unknown, with some studies suggesting the involvement of immunological, hormonal, genetic, and environmental factors [4]. Given that treatment options and outcomes differ for Rhupus syndrome compared to RA or SLE alone, it is vital to categorize patients accordingly [5]. Notably, the occurrence of black hairy tongues, typically associated with factors such as poor oral hygiene, certain medications, and smoking, has not been previously described in either RA or SLE [6].

## Case presentation

**Figure 1:**
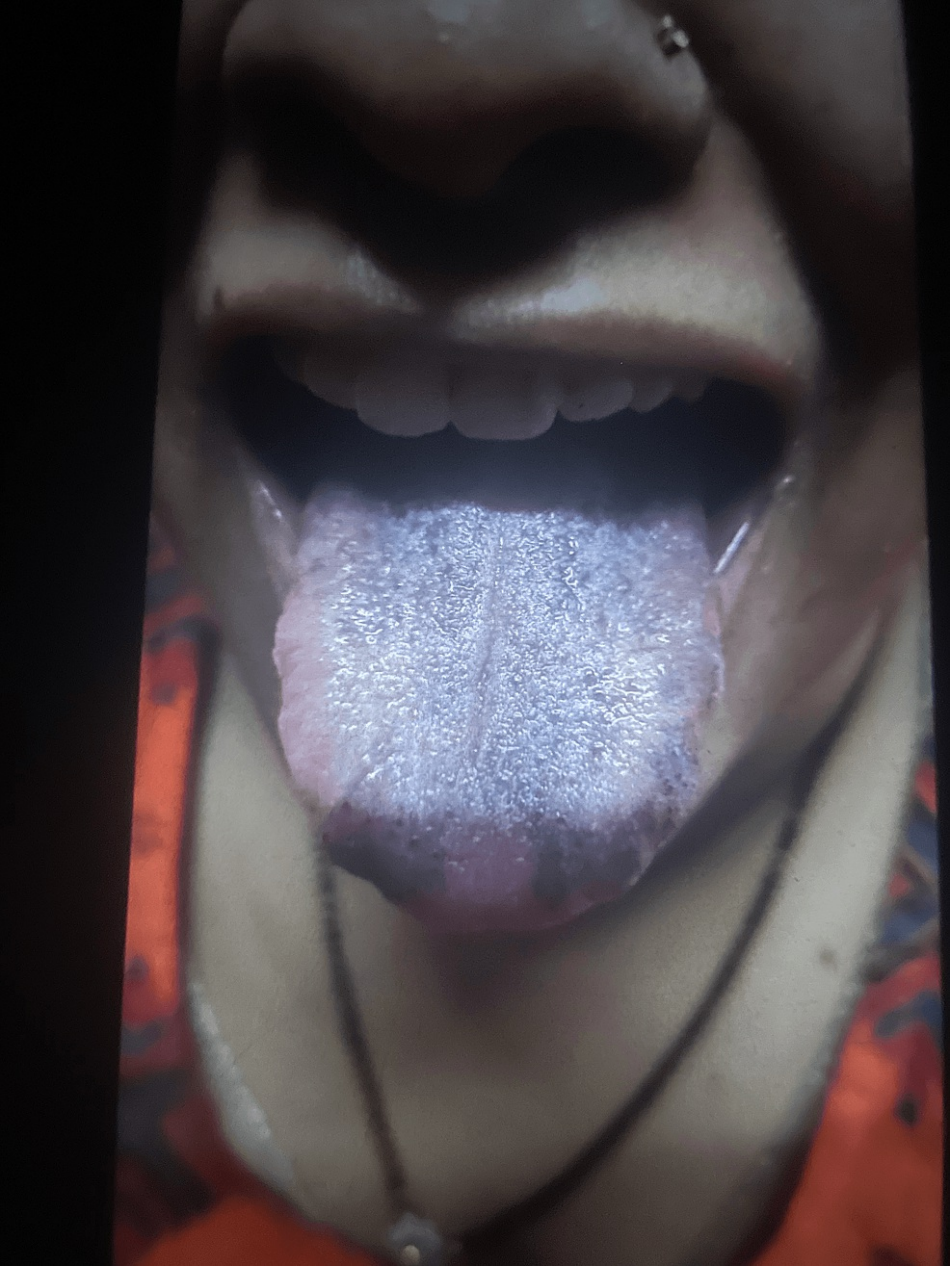
Appearance of the blackened tongue before treatment

Our patient is a 30-year-old female who does not smoke or consume alcohol. She follows a vegetarian diet and works as a homemaker. She was referred to our hospital from a primary care center due to her complaint of tongue blackening, which she had been experiencing for the past two to three weeks. Before seeking medical attention, she had tried to improve her oral hygiene, used mouth rinses and washes, and even underwent antibiotic therapy. Aside from the tongue blackening, the patient did not report any other complaints. Figure 1 illustrates the appearance of the blackened tongue when the patient initially presented to us.

**Figure 2:**
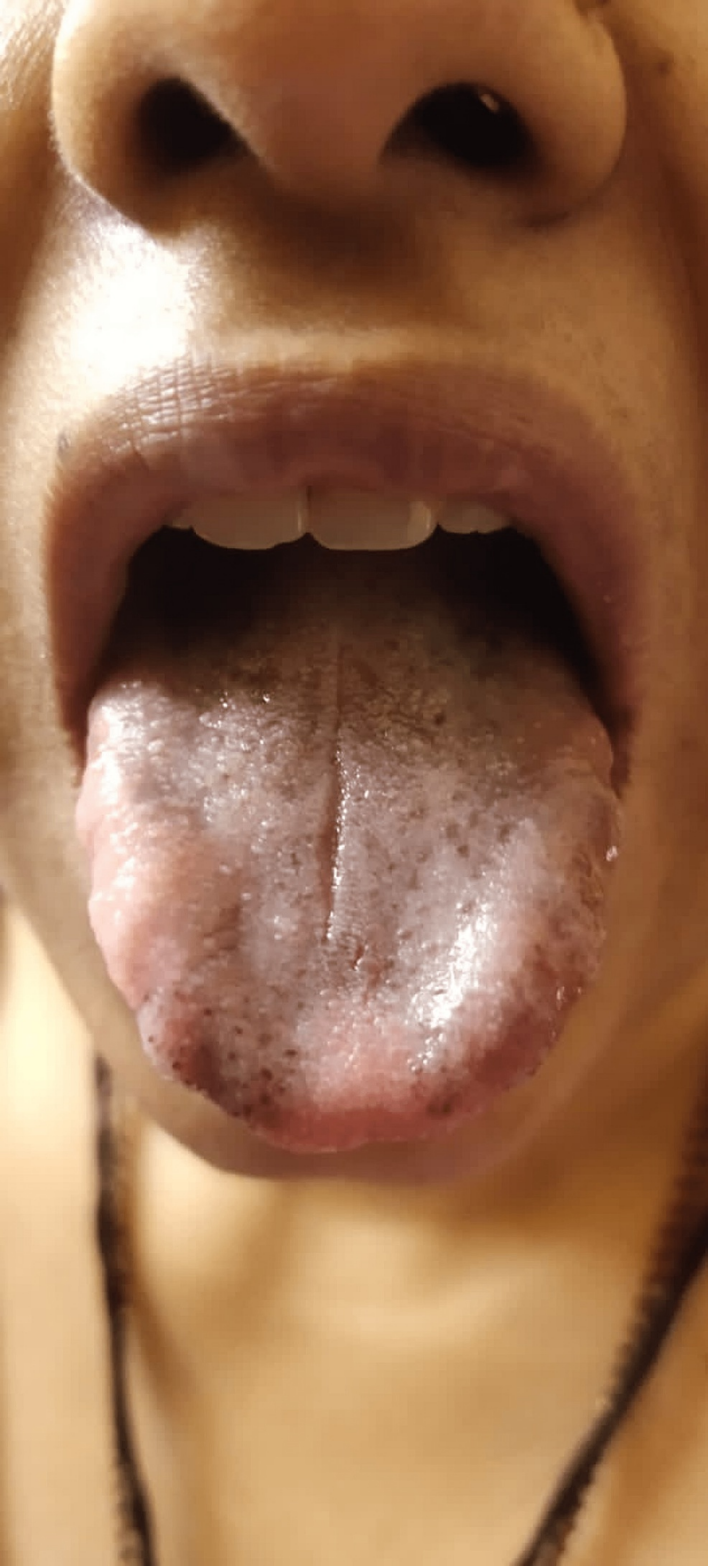
Post-treatment (follow-up after two weeks)

We assured her that the blackening of her tongue was a benign condition. The doctor prescribed her antifungal medication for the next two weeks and instructed her to return for a follow-up visit after that time. However, there was little to no improvement in her symptoms and only a minimal change in the color of her tongue. Figure 2 illustrates the negligible difference in color after approximately two weeks of antifungal treatment.

Upon further inquiry regarding her medical history, she disclosed that she had undergone an abortion in 2015 and subsequently gave birth to two children in 2017 and 2019. She experienced intermittent joint pains and mild swelling in her fingers, which she managed effectively with over-the-counter acetaminophen. Additionally, she mentioned having recurrent bouts of fever and facial redness whenever she ventured outdoors. We went ahead and did the basic blood tests, which were liver functions test with a few mild derangements (Table 1).

**Table 1:** Liver function tests A/G ratio: Albumin/Globulin ratio.

Test name	Result	Normal Reference Range
Total protein	9.1 gm/dl	6.5-7.8
Albumin	4.3 g/dl	3.9-5.0
Globulin	4.8 gm/dl	2.0-3.5
A/G ratio	0.9 ratio	1.5-2.5
Gamma-glutamyl transferase	50 U/L	12-38

The kidney function test had minor derangements (Table 2).

**Table 2:** Kidney function tests

Test name	Result	Normal reference range
Blood urea	18 mg/dl	15-36
Blood urea nitrogen	8.41 mg/dl	7-17
Creatinine	0.6 mg/dl	0.5-1.04
Uric acid	2.7 mg/dl	2.5-6.2
Calcium	9.3 mg/dl	8.4-10.2
Phosphorus	4.9 mg/dl	2.5-4.5
Sodium	139 mmol/L	137-145
Potassium	4.5 mmol/L	3.5-5.1
Chloride	107 mmol/L	98-107

CBC showed low hemoglobin and changes in the values of indices (Table 3). 

**Table 3:** Complete blood count. MCH: Mean corpuscular hemoglobin; MCV: Mean corpuscular volume; MCHC: Mean corpuscular hemoglobin concentration; RDW-CV: Red cell distribution width; ESR: Erythrocyte sedimentation rate.

Test name	Result	Normal Reference Range
Hemoglobin	9.7 gm/dl	12.0-15.0
Hematocrit	31.0%	36-46
MCV	71.5 fl	83-101
MCH	22.4 pg	27-32
MCHC	31.3 gm/dl	31.5-34.5
RDW-CV	16.8%	11.5-14.5
ESR	45 mm/hr	<20
C-reactive protein	8.7 mg/dl	<5.0

The lipid profile showed significant changes in the values of triglyceride, VLDL cholesterol, and non-HDL cholesterol. Thyroid profile showed minor changes; and antibody tests for SLE and RA showed significant changes as well (Table 4).

**Table 4:** Test results for lipid profile, thyroid function tests, and antibody tests for RA and SLE VLDL: Very low-density lipoprotein; Non-HDL: Non-high-density lipoprotein; Anti-DS DNA: Anti-double-stranded DNA; RA factor: Rheumatoid factor; Anti-CCP: Anti-cyclic citrullinated peptide.

Test name	Result	Reference
Lipid profile		
Total cholesterol	265 mg/dL	147-266
Triglyceride	412 mg/dL	35-212
VLDL cholesterol	75 mg/dL	5-40
Non-HDL cholesterol	212 mg/dL	<130
Thyroid function tests		
Free triiodothyronine (FT3)	2.56 pg/mL	2.77-5.27
Free thyroxine (FT4)	1.07 ng/dL	0.78-2.19
Thyroid-stimulating hormone (TSH)	5.35 MIU/L	0.46-4.68
Antibody tests for SLE		
Anti-nuclear antibody	10.1 AI	<1.5
Anti-DS DNA antibody	61.30 IU/ mL	<30.00
Antibody tests for RA		
RA factor	224.8 IU/mL	<12.0
Anti-CCP	>200.00 U/mL	<5.0

**Figure 3:**
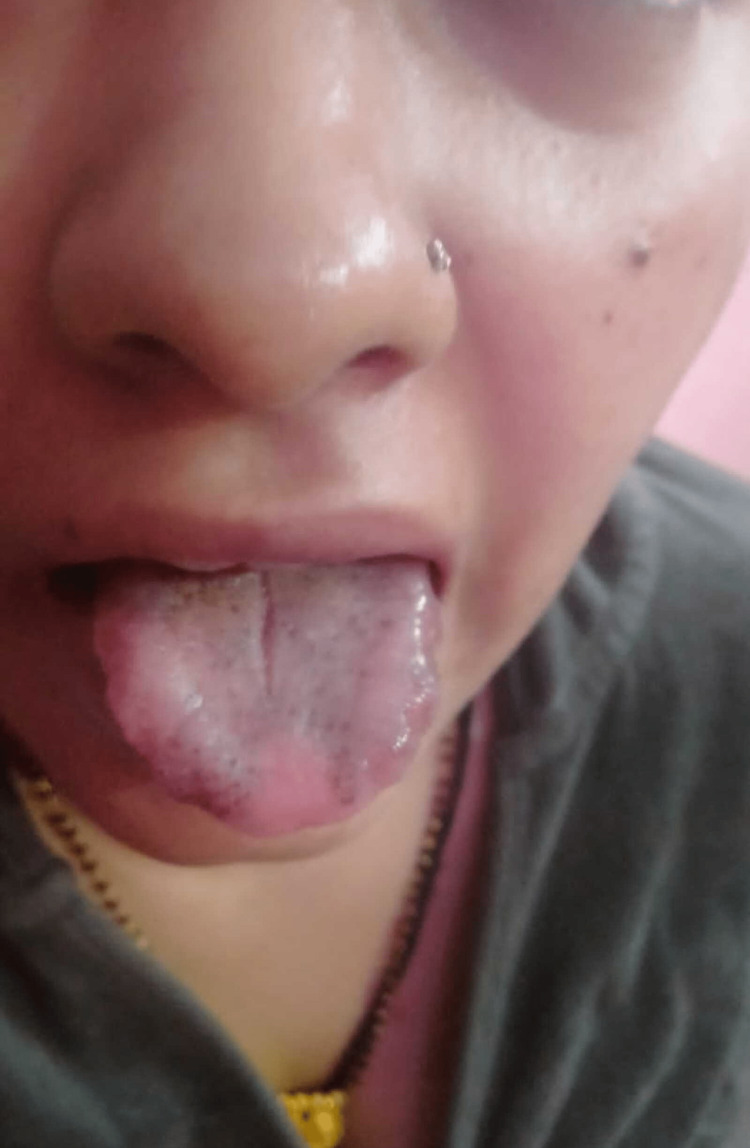
Follow-up after two months

Based on her clinical presentation and test results, we initiated treatment with hydroxychloroquine, thyroxine, methotrexate, and methylprednisolone. Additionally, we prescribed vitamin D and calcium supplements for two months. Figure 3 illustrates the color change observed in her tongue after two months of the prescribed treatment.

## Discussion

Rhupus syndrome, a rare condition, represents an overlap between SLE and RA, characterized by distinct clinical features and laboratory findings. Evidence supporting the diagnosis of Rhupus syndrome in patients with SLE and RA is limited [7]. Doctors usually treat Rhupus syndrome with steroids and DMARDs, medications commonly used for RA and SLE. When organ systems are affected, doctors may prescribe biological agents and immunosuppressants. However, the term “Rhupus syndrome” remains controversial due to the opposing immunopathological processes involved in SLE (characterized by abnormal activation of Th2 cytokines) and RA (involving Th1 response) [8]. Consequently, the incidence of this overlap between RA and SLE in arthritis patients is low, ranging from 0.01% to 0.2%. Some authors argue that Rhupus syndrome represents severe articular involvement in lupus rather than a mere overlap of SLE and RA [9].

Rhupus syndrome exhibits mild features associated with SLE, such as hematological abnormalities and skin and mucosal involvement. The patient presented with decreased hemoglobin, reduced white blood cell count, platelets, and intermittent oral ulcers. Elevated levels of inflammatory markers were observed, consistent with the high titers found in Rhupus patients. Our case also exhibited high levels of RA factor and anti-CCP antibodies, along with elevated ANA and anti-DS DNA antibodies [10]. The incidence of malar rash, hemolytic anemia, and involvement of the neurological and renal systems is lower in Rhupus syndrome compared to SLE. Rhupus syndrome rarely presents with nephrotic syndrome and renal insufficiency. The administration of methylprednisolone pulse therapy and initial corticosteroid dosages are typically lower in Rhupus syndrome compared to SLE flares (SLEDAI scoring is used to assess disease activity in SLE).

A study revealed an increased frequency of HLA-DR1 and HLA-DR2 alleles in patients with Rhupus syndrome [11,12]. Additionally, some authors have suggested that rheumatoid nodules in SLE patients may increase the risk of developing Rhupus syndrome [12]. Moreover, researchers have proposed that anti-CCP antibodies play a significant role in SLE patients with an elevated risk of developing erosive arthritis. These antibodies are thought to contribute to the clinical features and overlap of SLE with RA, referred to as Rhupus syndrome [13]. Radiological investigations have shown juxta-articular bone demineralization and joint erosion, which are part of the diagnostic criteria for RA.

Despite our patient experiencing mild joint pains and inconclusive x-ray results, we took proactive measures by prescribing methotrexate and folic acid. In her case, we based this decision on the elevated levels of RA factor and anti-CCP antibody [14].

## Conclusions

Rhupus syndrome is a significant clinical diagnosis as it differs in prognosis and treatment from SLE and RA. Given the limited data available on the condition, it is crucial to establish diagnostic criteria and develop a standardized treatment protocol. Currently, treatment strategies rely on the clinician's experience due to the absence of validated protocols. Since Rhupus syndrome can present mild symptoms such as a black tongue, it is essential to proactively address the condition to minimize patient morbidity. Timely diagnosis allows for early initiation of treatment, providing an advantage in preventing complications. Although rare, early detection can significantly influence patient outcomes and reduce the risk of severe consequences associated with delayed intervention.
